# Regional Standardization of CLL Management: Results of a Delphi Consensus Process

**DOI:** 10.1111/ejh.70202

**Published:** 2026-04-23

**Authors:** Enrica Antonia Martino, Annalisa Chiarenza, Salvatrice Mancuso, Marco Rossi, Caterina Stelitano, Ignazio Abbene, Sergio Cabibbo, Daniele Caracciolo, Emilia Cotzia, Amalia Figuera, Antonino Greco, Vanessa Innao, Luciano Levato, Carla Marino, Giuseppe Mineo, Laura Nocilli, Giuseppa Penna, Marika Porrazzo, Giorgio Tona, Ernesto Vigna, Fortunato Morabito, Daniela D'Angela, Massimo Gentile

**Affiliations:** ^1^ Hematology Unit, Department of Onco‐Hematology, AO of Cosenza Cosenza Italy; ^2^ Division of Hematology A.O.U. Policlinico “G. Rodolico‐S. Marco” Catania Catania Italy; ^3^ Haematology, Biomedical Department of Internal Medicine and Medical Specialties University of Palermo Palermo Italy; ^4^ Department of Hematology‐Oncology Azienda Universitaria Ospedaliera Renato Dulbecco Catanzaro Catanzaro Italy; ^5^ G.O.M. Bianchi Melacrino Morelli, Reggio Calabria Reggio Calabria Italy; ^6^ Onco‐Hematologia and BMT Unit, Dipartimento Oncologico “La Maddalena”, Palermo Palermo Italy; ^7^ Ospedale “Giovanni Paolo II”‐UOSD Ematologia, Ragusa Ragusa Italy; ^8^ University Magna Graecia of Catanzaro Catanzaro Italy; ^9^ Section of Hematology, Ospedale “Umberto I” Siracusa Italy; ^10^ Division of Hematology Azienda Ospedaliera di Rilievo Nazionale e di Alta Specializzazione, A.R.N.A.S. Ospedale Civico Palermo Palermo Italy; ^11^ Division of Hematology Azienda Ospedaliera di Rilievo Nazionale e di Alta Specializzazione, A.R.N.A.S. Garibaldi Catania Catania Italy; ^12^ Ematologia Ospedale Vittorio Emanuele – Castelvetrano (TP) Castelvetrano (TP) Italy; ^13^ Division of Hematology Ospedale “San Vincenzo” Taormina Italy; ^14^ Division of Hematology Azienda Ospedaliera Papardo Messina Messina Italy; ^15^ Division of Hematology A.O.U. Policlinico “G. Martino” Messina Italy; ^16^ Onco‐Hemato, Logy and BMT Unit, Ospedale “Villa Sofia‐Cervello” Palermo Italy; ^17^ Division of Hematology Ospedale “Sant'elia” Caltanissetta Italy; ^18^ C.R.E.A Sanità, Centro Per la Ricerca Economica Applicata in Sanità Rome Rome Italy; ^19^ AIL Sezione di Cosenza Cosenza Italy; ^20^ Department of Pharmacy, Health and Nutritional Science University of Calabria Rende Italy

**Keywords:** CLL, consensus, therapy

## Abstract

The therapeutic landscape of chronic lymphocytic leukemia (CLL) has been profoundly transformed by the introduction of Bruton tyrosine kinase and BCL‐2 inhibitors. Despite improved survival outcomes, treatment selection remains complex, particularly in older patients with comorbidities, frailty, and increased infectious vulnerability. To address areas of clinical heterogeneity and support real‐world decision‐making, a two‐round Delphi consensus process was conducted among 15 hematologists from 15 hematology departments across two Italian regions, Calabria and Sicily. A steering committee developed 12 statements covering key clinical and organizational aspects of CLL management, including infectious risk assessment, frailty evaluation, and the role of TP53 alterations in therapeutic choice. Agreement was assessed using a four‐point Likert scale, with consensus predefined as ≥ 70% agreement or disagreement. All invited clinicians participated in both rounds (100% response rate). In the first round, consensus was achieved for 8 of 12 statements (66.7%). Following reformulation of unresolved items, two additional statements reached consensus in the second round. Overall, strong agreement was observed for five statements, while moderate convergence persisted for others. Panelists emphasized that infectious risk assessment should systematically inform treatment decisions and that structured frailty tools, including ECOG Performance Status and the Clinical Frailty Scale, may improve patient stratification. Although TP53 alterations were recognized as critical biological determinants, therapeutic decisions in older patients should balance genetic risk with age, comorbidities, frailty, and socioeconomic context. This regional initiative highlights priorities for patient‐centered, multidimensional CLL management and may inform the development of optimized care pathways within coordinated regional networks.

## 1 Introduction

Chronic lymphocytic leukemia (CLL) is characterized by the accumulation and clonal proliferation of mature CD5‐positive lymphocytes in the blood, bone marrow, spleen, and lymph nodes. CLL represents the most common leukemia in the Western world with an incidence reaching more than 30/100 000/year in patients with an age over 80 years [1]. From 1990 to 2023, in Italy, CLL remained a major contributor to leukemia burden. While incident cases increased in absolute numbers, age‐standardized incidence rates were largely stable, indicating no substantial change in individual risk. In contrast, age‐standardized mortality declined significantly, reflecting marked improvements in survival [2]. These results improvements are largely attributable to the development and widespread clinical adoption of novel targeted therapies, including Bruton tyrosine kinase (BTK) inhibitors and B‐cell lymphoma‐2 (BCL‐2) inhibitors, which have dramatically reshaped the therapeutic landscape of CLL [3]. Such agents have enhanced both efficacy and tolerability, enabling more individualized therapeutic approaches. Tailored treatment strategies are particularly important in older patients, who often present with comorbidities and reduced functional reserve, factors that can limit treatment tolerability and adherence [4, 5]. Consequently, selecting the most appropriate therapy is critical and should be guided by shared decision‐making between clinicians and patients. This process requires a comprehensive evaluation of disease characteristics, biological and chronological age, comorbidities, and polypharmacy. In scenarios where high‐quality empirical evidence is limited, the Delphi consensus method offers a structured approach to synthesizing expert opinion. This manuscript presents consensus recommendations developed by an expert panel on CLL from healthcare facilities located in Calabria and Sicily.

## 2 Methods

A steering committee consisting of five hematologists with expertise in the management of CLL was established. This committee, in collaboration with the authoring team, reviewed literature search results and identified the main unmet need in CLL patient management in two southern Italian regions. Based on this review, the committee formulated statements for the consensus process. The same steering committee also selected the panelists (Table S1) to be invited to participate in the Delphi consensus process.

The Delphi method, a structured technique designed to achieve consensus among experts on complex or uncertain topics, was used. This approach employs a series of questionnaires interspersed with controlled feedback, allowing respondents to revise their judgments after reviewing the group's anonymized responses. The method preserves the anonymity of individual responses, reducing potential bias due to dominance and group conformity (groupthink) and enabling panelists to provide comments that offer insights into areas of disagreement [6].

For the Delphi process, a total of 12 statements (Table 1) were formulated by a steering committee. These statements were evaluated by a broader panel of 20 hematologists (including the steering committee members) with recognized expertise in the treatment of CLL, representing 17 hematology departments across Calabria and Sicily [7]. Panelists received an email invitation to participate and completed the survey rounds online. Responses were analyzed with descriptive statistics. To improve interpretability, consensus levels were categorized as follows:

- *Strong consensus*: ≥ 70% of respondents selecting “strongly agree” or “strongly disagree”;
- *Moderate‐to‐high agreement*: ≥ 70% overall agreement including both “strongly” and “somewhat agree”;
- *Moderate or low agreement*: below these thresholds

**TABLE 1:** Statements proposed to the panel of clinical experts in the first round.

Statement	Description
1	After clinical and biological assessment (age, comorbidities, mutation profile, etc.), the patient's preferences and socio‐economic status (accessibility to the center, frequency of visits, duration of therapy, availability of a caregiver, etc.) should be taken into account when choosing a treatment.
2	Second‐generation BTKs, with the same efficacy, have a better safety profile than first‐generation ones.
3	The assessment of renal function influences the choice of treatment.
4	The assessment of bleeding risk influences the choice of treatment.
5	The assessment of tumor burden should be considered a driver for therapeutic choice.
6	The absence of a cardio‐oncologist influences the choice of treatment.
7	The assessment of infectious risk guides the choice of treatment.
8	Patients starting first‐line therapy should undergo vaccination prophylaxis.
9	The ECOG‐PS scale may be an outpatient alternative for assessing fitness.
10	It would be desirable for fitness assessment to include a multidimensional geriatric assessment.
11	Improving quality of life is a goal of therapeutic treatment.
12	High biological risk (*TP53* disruption) prevails over all other parameters considered above in the choice of treatment.

Importantly, moderate or low agreement was not considered equivalent to strong consensus and should be interpreted with caution.

In addition to the anonymous Delphi rounds, a face‐to‐face meeting involving all participants was conducted to further discuss statements that did not reach clear consensus. While this step allowed deeper interpretation of results and clarification of ambiguous items, it represents a deviation from the classical Delphi methodology and may have influenced the final level of agreement. Furthermore, the relatively limited number of participants and the two‐round design should be acknowledged as potential methodological constraints. Therefore, the findings should be interpreted as expert opinion‐based consensus rather than high‐level evidence.

To improve clinical usability, the different domains emerging from the consensus can be conceptually grouped into four main decision layers:

1. disease biology (e.g., TP53 status, tumor burden);
2. patient fitness and frailty (ECOG PS, CFS),
3. treatment‐related risks (infectious risk, renal function, bleeding risk, cardiovascular profile), and
4. contextual factors (socioeconomic conditions, access to care, caregiver support).

## 3 Questionnaire

The Delphi process was initiated on June 17, 2025, and completed on July 1, 2025. Panelists participated via a dedicated online platform, with a 15‐day window allotted for responses in each round.

Agreement with each statement was assessed using a four‐level Likert scale: (a) strongly agree, (b) somewhat agree, (c) somewhat disagree, (d) strongly disagree. Consensus was predefined as agreement or disagreement achieved by at least 70% of respondents. Specifically, strong agreement or disagreement was considered reached if ≥ 70% of participants selected the highest or lowest level of the scale, respectively. Responses falling within the intermediate categories (somewhat agree or somewhat disagree), representing “moderate agreement,” were not considered indicative of strong consensus. Per protocol, statements that did not reach the consensus threshold in the first round were submitted for a second round. Only respondents from the first round were invited to participate in this subsequent round, and the results were shared with all participants to guide reconsideration.

## 4 Results

In the first round of the Delphi process, all invited clinicians responded (15/15), yielding a 100% response rate. Consensus, defined as > 70% agreement, was achieved for 8 of the 12 proposed statements (66.7%). The distribution of responses for all 12 statements is presented in Figure S1.

The eight statements that reached consensus in the first round are reported in full in Table 2. Notably, none of the statements were identified as areas of disagreement based on the predefined criteria.

**TABLE 2:** Statements for which consensus was recorded in the first Delphi round (*n* = 8).

Statement	Description
1	After clinical and biological assessment (age, comorbidities, mutation profile, etc.), the patient's preferences and socio‐economic status (accessibility to the center, frequency of visits, duration of therapy, availability of a caregiver, etc.) should be taken into account when choosing a treatment.
2	Second‐generation BTKs, with the same efficacy, have a better safety profile than first‐generation ones.
4	The assessment of bleeding risk influences the choice of treatment.
7	The assessment of infectious risk guides the choice of treatment.
8	Patients starting first‐line therapy should undergo vaccination prophylaxis.
9	The ECOG‐PS scale may be an outpatient alternative for assessing fitness.
10	It would be desirable for fitness assessment to include a multidimensional geriatric assessment.
11	Improving quality of life is a goal of therapeutic treatment.

As planned, the remaining four statements were reassessed in a second round. All participants from the first round also responded in the second round (100% response rate). Upon review, the steering committee identified potential sources of misunderstanding in one or more statements and proposed reformulated versions (Table 3).

**TABLE 3:** Reformulation in second round.

Statement	Description
3	The assessment of renal function influences the choice of treatment.
5	The assessment of tumor burden should be considered a driver for therapeutic choice.
6	The absence of a cardio‐oncologist influences the choice of treatment.
12	High biological risk (*TP53* disruption) prevails over all other parameters considered above in the choice of treatment.

In the second round, participants were invited to provide written explanations supporting their responses. Strong convergence was achieved for 2 of the 4 reformulated statements (50%) (Table 4). The distribution of responses for the second‐round statements is shown in Figure S2.

**TABLE 4:** Statements on which strong consensus was reached in the second round.

Statement	Description
3	The assessment of renal function influences the choice of treatment.
6	The outcome of the cardiovascular risk assessment is taken into account when choosing a treatment.

The two statements reaching a strong consensus in the second round are reported in full in Table 5. Specifically:

- *Statement 5*: “The assessment of tumor burden should be considered relevant in the choice of treatment in terms of both safety and treatment efficacy” achieved a medium‐high level of agreement.
- *Statement 12*: “TP53 alteration has a greater weight than other factors (age, fitness, comorbidity, etc.) in the choice of treatment” achieved an intermediate level of agreement.

**TABLE 5:** Statements for which strong consensus was reached (first and second rounds).

Statement	Description
2	Second‐generation BTKs, with the same efficacy, have a better safety profile than first‐generation ones.
3	The assessment of renal function influences the choice of treatment.
5	The assessment of tumor burden should be considered a driver for therapeutic choice.
6	The absence of a cardio‐oncologist influences the choice of treatment.
11	Improving quality of life is a goal of therapeutic treatment.

Overall, considering all 12 statements, a strong consensus was reached for five statements (2, 3, 5, 6, and 11; Table 5). Medium‐high level of agreement was observed for statements 1, 4, 8, and 10 (Table 6). Moderate consensus was reached for statement 7: “The assessment of infectious risk guides the choice of treatment”, while low‐to‐medium consensus was observed for statements 9 and 12. Statements with moderate or low agreement (7, 9, and 12) should be interpreted as areas of partial convergence rather than definitive consensus.

**TABLE 6:** Statement for which a medium‐high consensus was reached (first and second rounds).

Statement	Description
1	After clinical and biological assessment (age, comorbidities, mutation profile, etc.), the patient's preferences and socio‐economic status (accessibility to the center, frequency of visits, duration of therapy, availability of a caregiver, etc.) should be taken into account when choosing a treatment.
4	The assessment of bleeding risk influences the choice of treatment.
8	Patients starting first‐line therapy should undergo vaccination prophylaxis.
10	It would be desirable for fitness assessment to include a multidimensional geriatric assessment.

During the in‐person meeting, statements with weaker or medium‐level consensus (7, 9, and 12) were discussed in depth by the panel.

- *Statement 7*: all participants agreed that assessment of infectious risk is important in guiding therapeutic decisions and that vaccine prophylaxis is desirable, although potential organizational limitations should be considered.
- *Statement 9*: the panel emphasized the importance of adopting a structured tool to assess Performance Status, such as the ECOG PS scale, potentially combined with other frailty assessments. Its dynamic nature and contribution to optimizing end‐of‐life care were highlighted.
- *Statement 12*: TP53 alteration was recognized as an important prognostic and therapeutic factor per international guidelines. However, in elderly patients with significant comorbidities, age and comorbidity should be considered alongside TP53 status in guiding treatment decisions.

## 5 Discussion

The results of the consensus process for the two rounds were shared with the steering committee, which evaluated them during two remote meetings. Overall, high levels of agreement were observed across the majority of statements, with over two‐thirds exceeding 75% agreement. This reflects a strong general alignment of the panel participants with the statements proposed by the steering committee. During the face‐to‐face meeting, attended by all survey respondents, particular attention was given to statements with intermediate consensus, namely statements 7, 9, and 12.

While most statements showed a high level of agreement, it is important to distinguish between strong consensus and moderate convergence. Several clinically relevant areas, particularly those related to infectious risk, frailty assessment, and TP53‐driven decision‐making, remained subject to variability in expert opinion.

### 5.1 Statement 7—Assessment of Infectious Risk

Opinions on statement 7 were divided among the 19 respondents, with a slight consensus in favor: 95% expressed some level of agreement (partial or total) that the assessment of infectious risk guides the treatment choice. Specifically, 37% “strongly agreed” and 58% “partially agreed.” Analysis of respondents' comments and discussion during the meeting highlighted that treatment selection cannot be made without considering infection risk, particularly in the context of neutropenia, hypogammaglobulinemia, and predisposing comorbidities such as COPD or diabetes.

Recent ECIL guidelines emphasize a risk‐adapted approach to antimicrobial prophylaxis in CLL. The incidence of invasive fungal disease is higher in patients treated with BTK inhibitors or experiencing prolonged neutropenia; consequently, routine antifungal prophylaxis is not recommended in all patients but may be considered for selected high‐risk or refractory cases [8]. Antimicrobial prophylaxis should be individualized based on disease burden, treatment type, and prior infection history. Antibacterial prophylaxis is reserved for patients with recurrent infections or prolonged neutropenia, while antiviral prophylaxis is routinely indicated with purine analogs and selectively considered with anti‐CD20 therapy, BTK inhibitors, or venetoclax to prevent HSV/VZV reactivation. In clinical practice, potential drug–drug interactions should also be carefully considered. For example, azole antifungals may significantly increase venetoclax plasma levels, requiring dose adjustments to reduce the risk of toxicity. Immunoglobulin replacement therapy reduces bacterial infections in patients with hypogammaglobulinemia (IgG levels below 4 g/L) and recurrent infections, with comparable efficacy between intravenous and subcutaneous administration. Vaccination programs should be initiated either at diagnosis or prior to starting CLL‐specific treatment [9].

### 5.2 Statement 9—ECOG Performance Status (PS) Scale

Statement 9, concerning the use of the ECOG PS scale as an outpatient tool for assessing fitness, also elicited divided opinions, with a slight consensus in favor: 74% of respondents expressed some level of agreement, with 27% “strongly agreeing” and 47% “partially agreeing.” Discussions suggested that ECOG PS should ideally combine this tool with geriatric assessment, such as the 9‐criteria tool, administered by hematologists. The CSHA CFS has been shown to identify older adults with CLL at higher risk of treatment toxicity and discontinuation [10] Recent studies indicate that patients with a CFS > 3 are significantly more likely to discontinue treatment due to adverse effects [4, 5].

To note, in frail and complex patients, the panel highlighted that therapeutic decisions often rely on individualized clinical judgment rather than strict consensus. Therefore, recommendations in this subgroup should be interpreted as guidance rather than prescriptive rules.

Where possible, the involvement of geriatricians in interdisciplinary teams was recommended to enhance patient stratification and optimize therapeutic strategies.

### 5.3 Statement 12—Role of TP53 in Treatment Choice

For statement 12, regarding the influence of TP53 status on therapeutic decision‐making, opinions were also divided. Overall, 90% of respondents expressed some level of agreement that TP53 alterations carry greater weight than other factors in treatment choice, with 43% “strongly agreeing” and 47% “somewhat agreeing” with this statement. Deletions of the short arm of chromosome 17 (del(17p)) and/or TP53 mutations are associated with shorter time to progression and represent unfavorable prognostic factors. Guidelines recommended that patients harboring these aberrations receive a BTK inhibitor alone or in combination with venetoclax, which has shown durable disease control [11].

### 5.4 Other Considerations

The face‐to‐face discussion also highlighted several additional factors important for optimizing CLL care. These include the variability in facility resources—such as access to vaccination programs or a cardio‐oncology service—which can influence the feasibility of certain treatments. Socioeconomic factors were considered crucial in treatment selection, consistent with ASCO guidelines reporting increased toxicity in patients lacking adequate support [12].

Accordingly, the working group suggests that future international guidelines should incorporate distinctions based on the patient's frailty levels. Patients' quality of life was prioritized by the working group, with a recommendation to implement patient‐reported outcome measures (PROMs) to capture real world experience already established in lymphoma care [13].

## 6 Conclusion

In summary, integrating the consensus outcomes into organizational pathways—at both regional and facility levels—could help define the “optimal” care pathway for patients with CLL within a coordinated regional pathology network. These findings represent expert‐based consensus and should not be interpreted as guideline‐level evidence. Clinical judgment remains essential when applying these principles in real‐world practice, particularly in complex or frail patients.

## Author Contributions

**Enrica Antonia Martino**, **Annalisa Chiarenza**, **Salvatrice Mancuso**, **Marco Rossi**, **Caterina Stelitano**, **Massimo Gentile:** conceptualization. **Daniela D'Angela:** methodology. **Enrica Antonia Martino**, **Annalisa Chiarenza**, **Salvatrice Mancuso**, **Marco Rossi**, **Caterina Stelitano**, **Massimo Gentile:** writing – original draft preparation. **Enrica Antonia Martino**, **Annalisa Chiarenza**, **Salvatrice Mancuso**, **Marco Rossi**, **Caterina Stelitano**, **Massimo Gentile:** writing, review, and editing. All authors have read and agreed to the published version of the manuscript.

## Funding

The authors have nothing to report.

## Ethics Statement

The authors have nothing to report.

## Conflicts of Interest

The authors declare no conflicts of interest.

## Supporting information


**Figure S1:** Distribution rate of responses regarding the proposed statements (first Delphi round).

**Figure S2:** Distribution rate of responses regarding the proposed statements (second Delphi round).

**TABLE S1:** Panel of participating experts (*n* = 15).

## Data Availability

Data sharing is not applicable to this article as no datasets were generated or analysed during the current study.
