# Supplementary figures and images for: Regional Standardization of CLL Management: Results of a Delphi Consensus Process
Source: Eur J Haematol. 2026 Apr 23;117(2):442–7. doi: 10.1111/ejh.70202 (PMC13326801; doi:10.1111/ejh.70202)

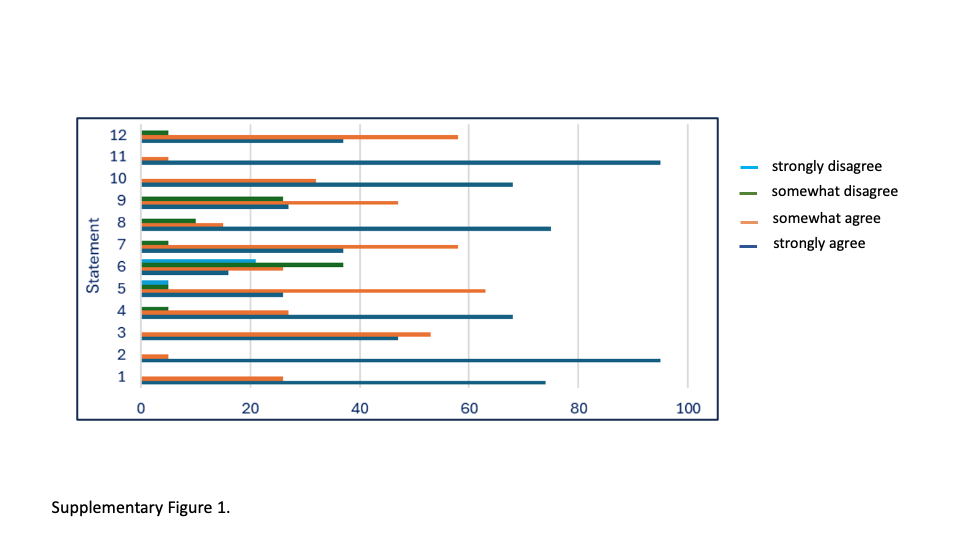

Supplement: Supplementary file 1 — Figure S1: Distribution rate of responses regarding the proposed statements (first Delphi round). [file EJH-117-442-s002.tiff]

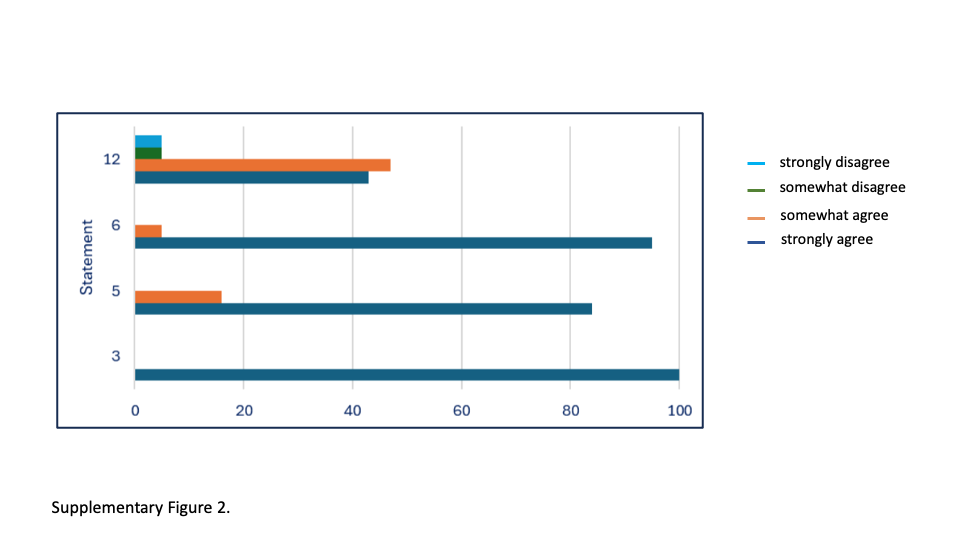

Supplement: Supplementary file 2 — Figure S2: Distribution rate of responses regarding the proposed statements (second Delphi round). [file EJH-117-442-s003.tiff]
