# Supplementary material for: Regional Standardization of CLL Management: Results of a Delphi Consensus Process
Source: Eur J Haematol. 2026 Apr 23;117(2):442–7. doi: 10.1111/ejh.70202 (PMC13326801; doi:10.1111/ejh.70202)
Supplement: Supplementary file 3 — TABLE S1: Panel of participating experts (n = 15). [file EJH-117-442-s001.docx]

**Supplementary Table 1**: Panel of participating experts (n = 15)

| **Delphi panel member** | **Specialty** | **Affiliation** |
| --- | --- | --- |
| Ignazio Abbene | Haematologist | Casa di Cura La Maddalena -Palermo |
| Sergio Cabibbo | Haematologist | Ospedale Giovanni Paolo II -Ragusa |
| Daniele Caracciolo | Haematologist | Policlinico Universitario -Catanzaro |
| Emilia Cotzia | Haematologist | Ospedale Umberto I - Siracusa |
| Amalia Figuera | Haematologist | Policlinico Universitario - Catania |
| Antonino Greco | Haematologist | ARNAS Civico - Palermo |
| Vanessa Innao | Haematologist | ARNAS Garibaldi - Catania |
| Luciano Levato | Haematologist | AOU Dulbecco - Catanzaro |
| Carla Marino | Haematologist | Ospedale Vittorio Emanuele – Castelvetrano (TP) |
| Enrica Antonia Martino | Haematologist | Azienda Ospedaliera Cosenza |
| Giuseppe Mineo | Haematologist | Ospedale S. Vincenzo – Taormina (ME) |
| Laura Nocilli | Haematologist | Azienda Ospedaliera Papardo - Messina |
| Giuseppa Penna | Haematologist | Policlinico Universitario - Messina |
| Marika Porrazzo | Haematologist | AOR Villa Sofia-Cervello - Palermo |
| Giorgio Tona | Haematologist | Ospedale Sant’Elia - Caltanissetta |
